# Plasma exchange in severe leptospirosis with multi-organ failure: a case report

**DOI:** 10.1186/1752-1947-7-169

**Published:** 2013-06-28

**Authors:** Dominic Taylor, Lazarus Karamadoukis

**Affiliations:** 1 Renal department, Dorset County Hospital NHS Foundation Trust, Williams Avenue, Dorchester DT1 2JY, UK

## Abstract

**Introduction:**

Leptospirosis presents with disease of variable severity; multi-organ failure can occur. In this situation, plasma exchange has been used with positive results, although the mechanism of action has not been fully explained.

**Case presentation:**

A 67-year-old Caucasian man developed severe leptospirosis with marked icterus, acute kidney injury and cardiorespiratory failure, after exposure to livestock. On the basis of previous reported cases, he was treated with plasma exchange. This led to a rapid improvement in his bilirubin level, cardiac and respiratory function, followed by renal function.

**Conclusion:**

We discuss the pathophysiology of the disease, and suggest that plasma exchange has a role in the treatment of severe sepsis caused by leptospirosis as well as immune complex-mediated organ injury.

## Introduction

Leptospirosis is caused by the *Leptospira* species of *Spirochaetes* and is presumed to be the world’s most common zoonosis. Infection usually follows contact with the urine of infected animals through broken or water-soaked skin or the conjunctivae. The largest reservoir of infection is rats, although leptospires have been isolated from cattle populations, and contact with livestock is an established risk factor [1]. In the United Kingdom there are around 60 serologically proven cases per year [2], although the incidence is far higher in tropical countries [1]. These are likely to be underestimates as many have mild or subclinical infection, and serological testing is imperfect and not always performed.

The disease follows a biphasic course [3]. An initial ‘infective’ or ‘septicemic’ phase lasts for 4 to 7 days during which leptospires can be found in the blood and the cerebrospinal fluid. Symptoms at this stage are non-specific and include fever, headache, myalgia, abdominal pain and uveitis. This is followed by a period of 1 to 3 days during which the fever resolves. An ‘immune’ phase follows, during which leptospires are excreted in the urine and anti-*Leptospira* immunoglobulin M (IgM) antibodies develop in the blood [1]. At this stage patients redevelop the initial symptoms, including fever and sometimes aseptic meningitis. Five to 10 percent of patients progress to the severe form of the disease which is also known as Weil’s disease [3]. This comprises jaundice due to hepatocellular dysfunction rather than hepatic necrosis and multi-organ involvement including acute kidney injury (AKI), pulmonary hemorrhage and adult respiratory distress syndrome.

Penicillins may shorten the duration of disease if given during the infective phase and have been shown to reduce urinary shedding of leptospires [4]. Treatment of severe disease after this stage is supportive, and may require intensive care support. There is also evidence of benefit from methylprednisolone in severe leptospirosis if given before the onset of multi-organ failure [5]. There is a considerable risk of death especially in cases with pulmonary involvement, where reported survival rates are as low as 16% [6]. However, there have been reports of the use of plasma exchange in such patients with positive outcomes [6-8]. We report successful treatment with plasma exchange in severe leptospirosis and discuss the possible mechanisms of action.

## Case presentation

A 67-year-old, male Caucasian livestock hauler presented to our acute medical unit with a 5-day history of lethargy and myalgia. He complained of dysuria with dark, malodorous urine, vomiting and hiccoughs. He was previously well with a history of bilateral inguinal hernia repair only. He took no regular medications but had been prescribed trimethoprim for presumed urinary tract infection 1 day previously.

On examination he was icteric, his temperature was 37.6°C and he was clinically dehydrated. Initial blood pressure (BP) was 118/65mmHg, heart rate 100 beats per minute. He had right upper quadrant abdominal tenderness but no evidence of peritonitis.

Initial blood tests revealed hemoglobin 78g/L (7.8g/dL), total white cell count 20×10^3^/μL, neutrophils, 18.3×10^3^/μL and platelet count 44×10^3^/μL. Coagulation parameters were in the normal range. Serum creatinine was 4.87mg/dL (431umol/L) and serum urea 63.9mg/dL (22.8mmol/L). Bilirubin was 12.9mg/dL (221μmol/L), alkaline phosphatase 236IU/L, and alanine transaminase (ALT) 152IU/L. C-reactive protein level was 280.2mg/L (2660mmol/L). Urine dipstick was positive for blood, protein and bilirubin and *Proteus mirabilis* was isolated on urine culture. Ultrasonography revealed normal appearances of the liver and bile ducts, and normal-sized kidneys with a non-obstructing left-sided staghorn calculus. Leptospirosis was considered as a cause of his illness. Serological testing was requested and he was treated initially with piperacillin-tazobactam.

Within 12 hours of admission he became hypotensive with BP 60/40mmHg, developed new onset atrial fibrillation and his temperature rose to 38.5°C. He remained hypotensive despite receiving 4.5L of intravenous fluid, and was transferred to the critical care unit. He required circulatory support with metaraminol up to 70mg/hour and later norepinephrine up to 0.3mg/hour. He developed hemoptysis and his respiratory function quickly deteriorated necessitating non-invasive ventilation. Despite circulatory support, he became anuric and continuous renal replacement therapy was initiated. He was given intravenous hydrocortisone 200mg followed by intravenous methylprednisolone 500mg and doxycycline was added.

**Figure 1:**
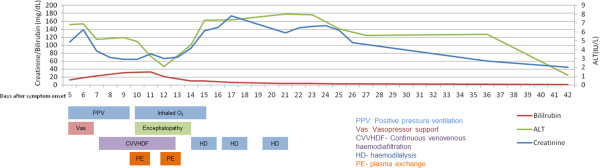
Changes in biochemical indices and critical care requirements with plasma exchange and renal replacement therapies.

After 48 hours, he developed marked hyperbilirubinemia with bilirubin 32.2mg/dL (550μmol/L) and fulminant liver failure with grade II hepatic encephalopathy. Chest radiography revealed diffuse alveolar edema. Blood film revealed no evidence of micro-angiopathic hemolytic anemia and the most probable diagnosis was thought to be leptospirosis, although serological evidence was not yet available. On the 5th day after admission, plasma exchange was initiated. Over the next 48 hours he underwent two 4-litre exchanges using a combination of fresh frozen plasma and human albumin solution. Biochemical parameters, timing of renal replacement therapy and plasma exchange, as well as respiratory and circulatory support requirements are shown in Figure 1. Renal biopsy was not performed due to thrombocytopenia and the increased risk of bleeding after plasma exchange.

After two plasma exchange sessions, there was a dramatic reduction of the serum bilirubin to 10.5mg/dL (180μmol/L) and of the ALT to 2.7mg/dL (47μmol/L). His mental state and respiratory function also improved significantly. It was possible to gradually withdraw vasopressors and respiratory support and the patient became oriented and alert. Further plasma exchange sessions were not deemed necessary owing to this dramatic improvement. At this point, serological testing was negative for hepatitis A, B, C and E, human immunodeficiency virus, Epstein–Barr virus, cytomegalovirus and *Borrelia burgdorferi*. Initial serology for *Leptospira* IgM was also negative.

Seven days after starting plasma exchange, he was transferred to our renal unit for intermittent hemodialysis, and after 9 days his renal function had recovered sufficiently to stop renal replacement therapy. After 14 days, *Leptospira* IgM was tested as positive, followed by a positive *Leptospira* microscopic agglutination test (MAT) tested 30 days after presentation. The patient remains well, with normal liver function tests and a serum creatinine of 1.52mg/dL (135umol/L).

## Discussion

This patient developed severe sepsis with AKI and multi-organ failure 5 to 7 days after the onset of symptoms of *Leptospira* infection, coinciding with the immune phase of the disease. Notably, he had pulmonary involvement which is a predictor of poor outcome [6].

The most common mechanism of renal injury in leptospirosis is tubulointerstitial nephritis with interstitial edema and a cellular infiltrate. This typically manifests as non-oliguric AKI, with hypokalemia caused by resulting tubular dysfunction [9]. In our case, there was oligo-anuria with no such electrolyte disturbance suggesting an alternative mechanism of AKI. As can be seen in Figure 1, initiation of plasma exchange led to an immediate improvement in serum bilirubin and ALT. Cardiovascular and respiratory function also improved dramatically allowing vasopressors and respiratory support to be withdrawn. His urine output improved 21 days after the onset of symptoms, followed by resolution of AKI.

There are other reports of severe leptospirosis treated successfully with plasma exchange. Tse *et al.*[7] described a patient with multi-organ failure including pulmonary infiltrates who improved dramatically after one plasma exchange only. Another patient was treated successfully with a series of five plasma exchanges, but suffered significant morbidity as a result of treatment complications [8]. Trivedi *et al.*[6] report a case series of 114 patients with leptospirosis complicated by pulmonary hemorrhage that were treated either with two plasma exchange sessions and one dose of cyclophosphamide, or with supportive treatment alone. The survival among those treated with plasma exchange was 77% compared with 17% in those treated with supportive treatment alone. The mechanism by which plasma exchange results in the observed clinical and biochemical improvement of patients with severe leptospirosis is not fully understood. We have outlined possible mechanisms below:

1) Plasma exchange may disrupt the mechanisms causing tissue damage in severe sepsis.

In the infective phase of leptospirosis, systemic inflammation can cause endovascular injury similar to that occurring in severe sepsis [9]. The outer membrane of *Leptospira* shares characteristics of both Gram-positive and Gram-negative bacteria. Its main components are phospholipids, outer membrane proteins (OMP) and lipopolysaccharides (LPS) that act as endotoxins [3]. In the bloodstream, the binding of LPS to toll-like receptors (TLR)4 on the surface of B lymphocytes results in the activation of B-cells and the production of IgM against the LPS of *Leptospira*[10]. Leptospiral IgM can opsonize the bacteria and promote their phagocytosis and killing by neutrophils and macrophages. *Leptospira* that survive this immune response are able to evade complement-mediated killing and quickly disseminate and colonize the liver and their other main target organs which are the lung and the kidney [3]. In the liver and kidneys, leptospiral LPS binds to TLR2 and TLR4 causing activation of B-cells and T-cells [10]. Activated B and T-cells secrete interferon-γ that promote the killing of *Leptospira*, but also produce pro-inflammatory cytokines such as interleukin-6 (IL-6) and tumor necrosis factor (TNF) leading to tissue inflammation [10]. TNF and IL-6 are also important mediators in the pathogenesis of meningococcal infection, but plasma exchange had minor or no effect in the plasma concentration of these cytokines in patients with meningococcal infection [11]. The evidence in the literature for the use of plasma exchange in sepsis is conflicting, because non-randomized clinical trials and case series report survival benefit, whereas two randomized controlled trials found no benefit [12].

2) Plasma exchange may prevent immune complex-mediated tissue injury.

Although vasculitis has been demonstrated in histological specimens from cases with severe leptospirosis, it has not been a persistent finding neither is it thought to be the primary pathological mechanism [13]. In the immune phase of the disease, there is evidence for the development of pathogenic immune complexes and the disappearance of these complexes coincides with improvements in liver and kidney function [14]. In murine models, activation of TLR by viral antigens can induce immune complex glomerulonephritis [15], but such a response has not been observed in leptospirosis. Instead, activation of TLR2 and TLR4 by LPS on renal epithelial cells is thought to cause tubulointerstitial nephritis [3,8]. In addition, leptospiral OMP has also been shown to cause accumulation of extracellular matrix in tubular cells contributing to the development of renal fibrosis [3].

The pathophysiology of lung injury in severe leptospirosis is also unclear and is thought to be induced either by unidentified toxins or by an autoimmune mechanism. The deposition of IgM, IgA, IgG and C3 in the alveolus of patients with pulmonary hemorrhage secondary to leptospirosis and in a guinea pig model of severe pulmonary leptospirosis is similar to the pattern seen in Goodpasture’s syndrome [3,9]. However, sera from patients with leptospirosis did not recognize the human glomerular basement membrane (GBM) and it is therefore unlikely that the lung disease seen in leptospirosis is caused by an anti-GBM antibody mechanism [3].

The serological diagnosis of leptospirosis remains challenging and in our case the diagnosis was made on the basis of the clinical picture. The available serological tests for *Leptospira* are an enzyme immunoassay (EIA), measuring leptospiral IgM, and a MAT. The EIA becomes positive from 5 days after infection, but can take longer, especially if antibiotics have been administered. Confirmation by MAT is recommended, which may be positive from the 10th day after the onset of symptoms [1].

## Conclusion

In summary, our patient had features of severe leptospirosis, with AKI and pulmonary involvement. Despite predictors for poor outcome, his clinical condition and biochemical parameters improved dramatically after two plasma exchanges. Although it is possible that the patient recovered spontaneously with supportive management, his dramatic improvement leads us to believe that plasma exchange had a vital role in his recovery. We hypothesize that plasma exchange prevents injury mediated by sepsis or by an unknown immune mechanism in severe leptospirosis. In our experience, serological testing was not helpful due to the long delay to a positive result and we recommend treatment based on the clinical picture. We advocate the use of plasma exchange in deteriorating severe disease necessitating intensive care support despite standard treatment with antibiotics.

## Consent

Written informed consent was obtained from the patient for publication of this case report and accompanying images. A copy of the written consent is available for review by the Editor-in-Chief of this journal.

## Competing interests

The authors declare that they have no competing interests.

## Authors’ contributions

LK led the care of the patient, wrote much of discussion, and led the project. DT reviewed the patient’s notes, produced the case report, introduction and figure. Both authors read and approved the final manuscript.

## References

[B1] Levett PN Leptospirosis Clin Microbiol Rev 2001 14 2 296 10.1128/CMR.14.2.296-326.2001 11292640 PMC88975

[B2] World Health Organisation http://www.who.int/zoonoses/diseases/leptospirosis/en/ accessed December 2011

[B3] Fraga TR Barbosa AS Isaac L Leptospirosis: aspects of innate immunity, immunopathogenesis and immune evasion from the complement system Scand J Immunol 2010 73 408 419 10.1111/j.1365-3083.2010.02505.x 21204903

[B4] Watt G Padre LP Tuazon ML Calubaquib C Santiago E Ranoa CP Laughlin LW Placebo-controlled trial of intravenous penicillin for severe and late leptospirosis Lancet 1988 1 8583 433 2893865 10.1016/s0140-6736(88)91230-5

[B5] Kularatne SA Budagoda BD de Alwis VK Wickramasinghe WM Bandara JM Pathirage LP Gamlath GR Wijethunga TJ Jayalath WA Jayasinghe C Pinto V Somaratne P Kumarasiri PV High efficacy of bolus methylprednisolone in severe leptospirosis: a descriptive study in Sri Lanka Postgrad Med J 2011 87 13 17 10.1136/pgmj.2009.092734 21106802

[B6] Trivedi SV Vasava AH Bhatia LC Patel TC Patel NK Patel NT Plasma exchange with immunosuppression in pulmonary alveolar haemorrhage due to leptospirosis Indian J Med Res 2010 131 429 433 20418558

[B7] Tse KC Yip PS Hui KM Li FK Yuen KY Lai KN Chan TM Potential benefit of plasma exchange in treatment of severe icteric leptospirosis complicated by acute renal failure Clin Vaccine Immunol 2002 9 2 482 484 10.1128/CDLI.9.2.482-484.2002 PMC119963 11874897

[B8] Cerdas-Quesada C Potential benefits of plasma exchange by apheresis on the treatment of severe Icteric Leptospirosis: Case report and literature review Transfus Apher Sci 2011 45 2 191 194 10.1016/j.transci.2011.07.012 21889407

[B9] Yang C-W Wu M-S Pan M-J Leptospirosis renal disease Nephrol Dial Transplant 2001 16 Suppl. 5 73 77 11509689 10.1093/ndt/16.suppl_5.73

[B10] Werts C Leptospirosis: a Toll road from B lymphocytes Chang Gung Med J 2010 33 6 591 601 21199604

[B11] van Deuren M Frieling JTM van der Ven-Jongekrijg J Neeleman C Russel FGM van Lier HJJ Bartelink AKM van der Meer JWM Plasma patterns of Tumour Necrosis Factor-α (TNF) and TNF soluble receptors during acute meningococcal infections and the effect of plasma exchange Clin Infect Dis 1998 26 918 923 10.1086/513933 9564476

[B12] Szczepiorkowski ZM Bandarenko N Kim HC Linenberger ML Marques MB Sarode R Schwartz J Shaz BH Weinstein R Wirk A Winters J Guidelines on the use of therapeutic apheresis in clinical practice-evidence-based approach from the apheresis applications committee of the American Society for Apheresis J Clin Apher 2007 22 106 175 10.1002/jca.20129 17394188

[B13] Medeiros FR Spichler A Athanazie DA Leptospirosis-associated disturbances of blood vessels, lungs and hemostasis Acta Trop 2010 115 155 162 10.1016/j.actatropica.2010.02.016 20206112

[B14] Galli M Esposito R Crocchiolo P Chermotti M Gasparro P Dall’Agio P Immune complexes in leptospirosis Infection 1985 13 156 10.1007/BF01642877 4030109

[B15] Pawar RD Patole PS Zecher D Segerer S Kretzler M Schlöndorff D Anders HJ Toll-like receptor-7 modulates immune complex glomerulonephritis J Am Soc Nephrol 2006 17 141 149 10.1681/ASN.2005121335 16280469

